# Modelling and simulation of a thermally induced optical transparency in a dual micro-ring resonator

**DOI:** 10.1098/rsos.170381

**Published:** 2017-07-12

**Authors:** Joseph Lydiate

**Affiliations:** University of Manchester, Electronic Engineering, Oxford Road, Manchester M13 9PL, UK

**Keywords:** optical, transparency, thermal, micro-ring, resonator, sensor

## Abstract

This paper introduces the simulation and modelling of a novel dual micro-ring resonator. The geometric configuration of the resonators, and the implementation of a simulated broadband excitation source, results in the realization of optical transparencies in the combined through port output spectrum. The 130 nm silicon on insulator rib fabrication process is adopted for the simulation of the dual-ring configuration. Two titanium nitride heaters are positioned over the coupling regions of the resonators, which can be operated independently, to control the spectral position of the optical transparency. A third heater, centrally located above the dual resonator rings, can be used to red shift the entire spectrum to a required reference resonant wavelength. The free spectral range with no heater currents applied is 4.29 nm. For a simulated heater current of 7 mA (55.7 mW heater power) applied to one of the through coupling heaters, the optical transparency exhibits a red shift of 1.79 nm from the reference resonant wavelength. The ring-to-ring separation of approximately 900 nm means that it can be assumed that there is a zero ring-to-ring coupling field in this model. This novel arrangement has potential applications as a gas mass airflow sensor or a gas species identification sensor.

## 1. Introduction

A coupled resonator-induced transparency (CRIT) may be introduced into the output spectrum of a micro-ring resonator by generating contra-rotating modes within the micro-ring resonator [1–3]. The theory of coupled atomic resonant systems using coherent optical media has been presented [4] and similarities can be seen between micro-ring resonator generated optical transparencies and electrically induced transparencies (EIT) generated in a gas phase at the atomic level. A coupled micro-ring resonant system has also been implemented into a Mach–Zehnder arrangement to realize an optical transparency in the combined Mach–Zehnder output spectrum [5]. In the novel approach in the current work, an optical transparency is thermally induced which does not rely on the ring-to-ring coupling coefficient strengths, adopted in the CRIT resonator, to achieve the optical transparency. This novel configuration is referred to by the acronym OTRR (optically transparent ring resonator). This allows for brevity when referring to this configuration in the text. In this paper, the difference between the CRIT and the OTRR is explained in the form of MATLAB spectral plots along with explanations of the distinct functional differences between the CRIT and the OTRR. The OTRR achieves the transparency by constructive and destructive interference at the combined output of the OTRR. The constructive and destructive interference is a consequence of the phase mismatch between the two resonators due to the applied heat changing the optical characteristics of the waveguides. Thermal stability is also an important consideration in the design of a micro-ring sensor. A change in the waveguide core temperature, due to an ambient temperature change, can manifest in the output as a spectral shift. This ambient temperature-induced spectral shift is, therefore, an unwanted noise component of the output of the sensor. A paper which addresses thermal noise in the micro-ring resonator [6], discusses the athermal operation of a micro-ring resonator. In the theoretical work of Deng *et al.* [6], the blue shift of the resonance splitting compensates for the red shift due to the thermal drift of the resonant split peak. In the design in [6], however, there is still a requirement for a negative thermo-optical coefficient material to be fabricated into the design to provide a thermal coupling coefficient magnification factor. The novel dual resonator design in this paper is configured such that any environmentally induced variations, such as temperature or structural change, affect the two resonators concurrently. The stimulus only affects one resonator so only the spectral difference in wavelength shift needs to be measured to infer the measurand. If thermal drift is presented as an issue, a brief outline of how thermal feedback could be applied to the OTRR to reduce thermal drift is given in §3.3. Application areas using the optical transparency in a micro-ring sensor are sparse if non-existent so two sensor applications are suggested and outlined in this work.

The first application is a mass flow sensor and the second suggested application is a gas species identification sensor. In these two applications, the waveguides are not exposed to the gases to be measured. The mass flow or gas species is inferred by a resonant wavelength shift induced by the heat transfer characteristics of the gas and the integrated heaters. The waveguides are not exposed to the gas and, therefore, they are not contaminated by particle constituents in the gas.

## 2. A novel dual-ring configuration

**Figure 1.**
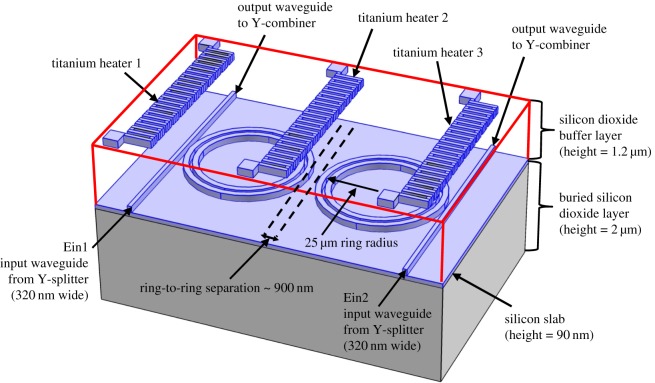
The OTRR configuration.

The novel dual-ring configuration of the OTRR is shown in figure 1. In high-contrast materials, losses due to smaller radius bends, become an issue. The slotted ring design was adopted for this model in order to reduce the bend loss in the rings and so confine the circulating fields to the central slots of the concentric rings. The arrangement of figure 1 is similar to a Mach–Zehnder configuration with the addition of two rings sufficiently isolated from one another so there is a zero-field coupling between the two resonators. The OTRR is also similar to a cascaded Vernier configuration [7], but in the configuration of reference [7] the two ring resonators are coupled. In contrast with [7], there is no offset between the two resonators in the OTRR, and there is also an additional input (Ein2, figure 1), which is not used in the resonator configuration of Claes *et al.* [7]. The OTRR is modelled on the silicon on insulator (SOI) 130 nm CMOS rib fabrication process with a silicon slab layer of 90 nm giving a total waveguide height of 220 nm and an input/output waveguide width of 320 nm. The SOI and rib fabrication processes are explained in [8]. The outer ring width is 250 nm, whereas the inner ring width is 290 nm. The silicon waveguides are fabricated on top of a 2 µm silicon dioxide (SiO_2_) layer. A 1.2 µm SiO_2_ buffer layer is then deposited on top of the silicon waveguides. There are two titanium nitride (TiN) heaters positioned above the ring coupling regions on top of the SiO_2_ buffer layer. A third heater is positioned above the central region of the rings.

The concentric rings form a slot separating the outer and inner rings. Each slotted ring has a mean diameter of 25 µm to the centre of the slot. The inputs, Ein1 and Ein2, originate from a 50/50 Y-Splitter and the Y-Splitter is fed from a grating coupler. The through port outputs are combined in a Y-combiner where the combined output can be extracted using a grating coupler. To give greater clarity and understanding to figure 1, the Y-splitter/combiner and grating couplers are omitted and an exaggerated aspect ratio is used for the figure.

## 3. Simulation and modelling methods

The finite-element method (FEM) using COMSOL and the thermo-optic multi-physics utility of FemSIM, were implemented to determine the temperature-dependent effective index and group effective index of the waveguides. The beam propagation method (BPM), using the BeamPROP thermo-optic multi-physics utility, was applied to determine the temperature-dependent through coupling coefficients and the absorption coefficients of the rings.

The author's computational resources were insufficient to execute a full three-dimensional (3D) multi-physics heat transfer Joule/current heating and photonic parametric model in COMSOL. A two part simulation method was therefore adopted by first using COMSOL, then applying the results from the 3D COMSOL simulations to the two-dimensional (2D) FemSIM and 3D BeamPROP models.

A 3D model of the input waveguide and slotted ring coupling arc section was constructed in COMSOL. A parametric scan using current densities over the range 1.111 × 10^9^ to 14.444 × 10^9^ A m^−2^ was applied to the TiN heater. These current densities correspond to an applied heater current over the inclusive range of 1–13 mA. The application of a current density range as a parametric sweep, allowed the temperature to be determined anywhere within the 3D model. The heater and waveguide core temperatures could then be determined using average volumetric temperature probes. It was required to establish the spatial influence of the heater at a maximum current density of 14.444 × 10^9^ A m^−2^ (13 mA). The heater exerted minimal influence over the refractive index (RI) at a distance of 8.15 µm from the centre of the input waveguide to the chord of the radius of the arc of the slotted ring. The latter simulation allowed for a partial 3D model to be constructed which resulted in a reduced demand on simulation resources. The simulated temperature profile of the waveguide cores was then examined to establish the extent of the heater influence on the waveguides. Heater voltages were also recorded over the applied range of heater current densities and these are given in table 14 (appendix A2). This novel resonator may see potential use as a sensor and so it is expedient to refer to one of the resonators as the sense resonator and the other resonator will be the reference resonator.

The resonator influenced by heater 1 is the sense resonator while the other resonator is the reference resonator. The central heater (heater 2), above the central resonator section was also modelled in COMSOL using the same current density parameters as those used for heater 1. These data were also used in producing the spectral plots of the OTRR output.

**Figure 2.**
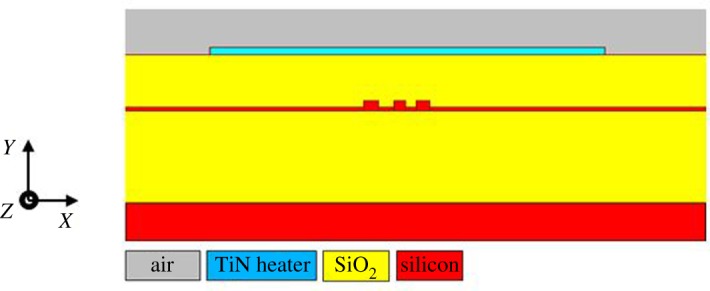
The 2D FEM model of the waveguide slotted ring coupling region.

Figure 2 shows the cross-section of the sense resonator at the mid-point position along the input waveguide *z*-axis. The heater temperatures determined from the 3D parametric COMSOL model was applied to the 2D FemSIM model of figure 2, to obtain the following contour plots and waveguide core temperature profile plots.

The model of figure 2 was also used to determine the temperature-dependent effective index, group effective index and ring loss data. The temperature-dependent coupling and ring absorption coefficients were determined by applying BeamPROP to a 3D model. The temperature-dependent coupling coefficients and ring absorption coefficients are given in table 1.

**Table 1.** Input guide-to-ring coupling coefficients and ring absorption coefficient as a function of heater current and temperature.

heater current	heater temperature (K)	coupling coefficient	ring absorption coefficient (cm^−1^)
0 mA	293.15	0.520	6.7
5 mA	369.9	0.495	4.4
7 mA	443.6	0.477	4.1
9 mA	541.8	0.439	3.9

**Figure 3.**
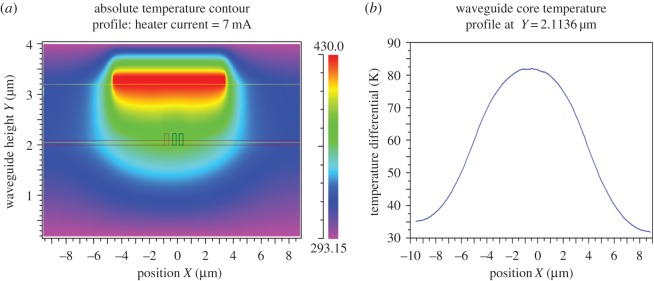
(*a*) Thermal contour plot (heater current = 7 mA). (*b*) Waveguide profile plot (heater current = 7 mA).

Figure 3*a*,*b* shows the 2D temperature contour plot and the waveguide core cross-section temperature profile plot for a simulated heater current of 7 mA. The temperature data extracted from the model of figure 2 are given in table 2 and the 2D data can be compared with the 3D data extracted from the COMSOL model.

**Table 2.** Comparison of the temperatures of the 3D and 2D micro-ring models.

heater temp = 443.6 K	heater current	outer ring core temperature	inner ring core temperature
3D COMSOL Model	7 mA	80.61 K above substrate	80.22 K above substrate
2D RSoft Model	7 mA	81.80 K above substrate	81.19 K above substrate

**Figure 4.**
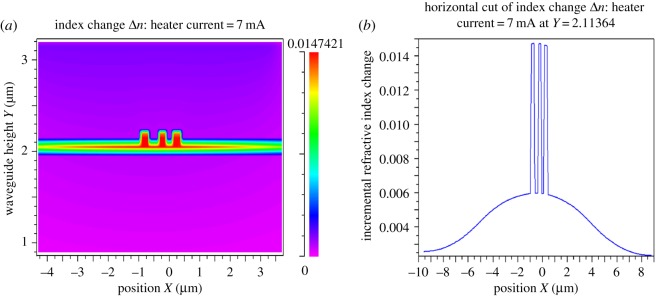
(*a*) RI contour plot (heater current = 7 mA). (*b*) Incremental RI profile plot (heater current = 7 mA).

Figure 4*a* shows the contour plot of the incremental RI increase for a simulated heater current of 7 mA. Figure 4*b* shows the cross-section incremental RI increase, at the central waveguide core *Y*-position of *Y* = 2.11 µm, for the same heater current of 7 mA.

Figure 4*a*,*b* implicitly indicate the concentration of heat at the waveguide cores. These figures also reveal that the silicon waveguides and slab are good heatsinking materials compared with the SiO_2_.

Table 3 provides the FemSIM multi-physics generated RI incremental data for heater 1 currents of 5, 7 and 9 mA.

**Table 3.** Input coupling region waveguide RI increments as a function of heater 1 current and temperature.

heater 1 current = 5 mA	heater 1 current = 7 mA	heater 1 current = 9 mA
heater 1 temperature = 369.9 K	heater 1 temperature = 443.6 K	heater 1 temperature = 541.8 K
RI increment = 0.007514	RI increment = 0.014719	RI increment = 0.024348

### 3.1. The effect of thermo-optic and linear expansion coefficients

The differential change in resonant wavelength, as a function of a change in waveguide core temperature [9], is given by the analytically derived equation

3.1 $$\Delta \lambda_{\mathrm{res}}=\frac{\lambda_{\mathrm{res}}}{n_{g0}}\;\left(\frac{\partial n_{\mathrm{eff}}}{\partial T}\;\text{d}T+n_{g0}\frac{1}{L}\;\frac{\partial L}{\partial T}\;\text{d}T\right),$$

λ_res_ is the unperturbed resonant wavelength, ∂*n*_eff_/∂*T* is the effective index thermo-optic coefficient of silicon, 1/*L* ∂*L*/∂*T* is the coefficient of linear expansion of silicon, *n*_g0_ is the unperturbed group effective index.

**Figure 5.**
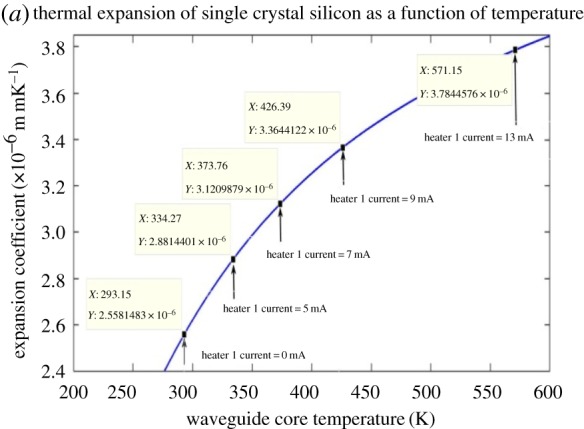
Thermal expansion of silicon.

**Figure 6.**
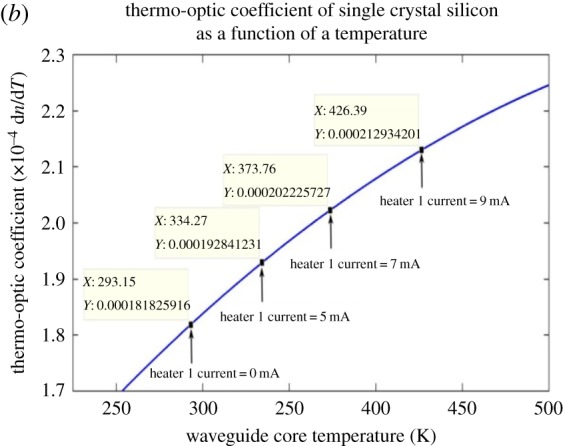
Thermo-optic dependency of silicon.

The thermo-optic and linear expansion coefficients are also a function of temperature and so figure 5 shows the thermal expansion coefficient of silicon as a function of temperature. Figure 6 also shows that the thermo-optic coefficient is also temperature dependent.

The thermal expansion coefficient data used to construct figure 5 were obtained from an empirically derived formula [10] and this formula is given as:

3.2 $$\beta_{\mathrm{si}}=s+Ax^{2}\frac{\exp(x)}{{\left(\exp(x)-1\right)}^{2}}\;+\;B\frac{{\left(y-1\right)}^{2}}{\left(1+hy\right)},$$

where *x *= 685/*T*, *y *= *T*/395, *s* = −0.687 × 10^−6^, *A* = 5 × 10^−6^, *B* = 0.22 × 10^−6^ and *T* is the absolute temperature. From the study of Padmaraju & Bergman [9], equation (3.2) may be used over the absolute temperature range of 90–850 K.

The thermo-optic coefficient data used to construct figure 6 were obtained from an empirically formulated data table [11]. The data in the table of Swenson [10] may be used over the absolute temperature range of 293–750 K.

Table 4 shows the incremental refractive index (ΔRI), of the waveguides, derived using the empirical data of Swenson [10].

**Table 4.** Incremental RI increments as a function of heater 1 current and temperature using empirically derived formula.

heater 1 current = 5 mA	heater 1 current = 7 mA	heater 1 current = 9 mA
heater 1 temperature = 369.9 K	heater 1 temperature = 443.6 K	heater 1 temperature = 541.8 K
RI increment = 0.007928	RI increment = 0.016300	RI increment = 0.028368

**Figure 7.**
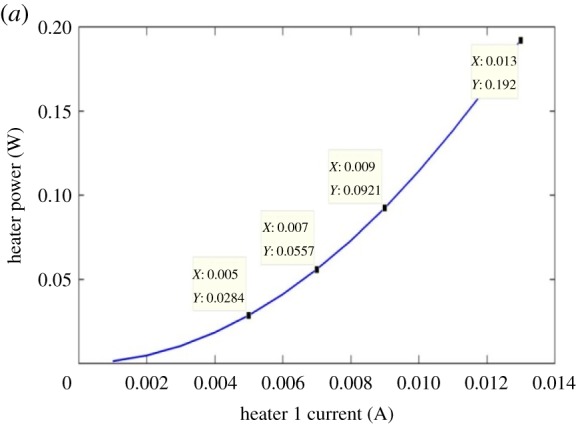
Heater power as a function of heater current.

**Figure 8.**
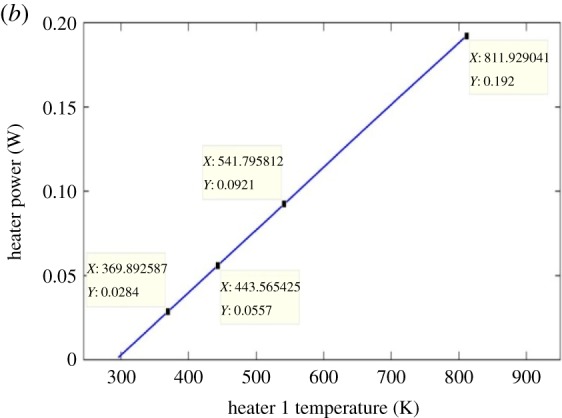
Heater power as a function of heater temperature.

Figure 7 shows the expected nonlinear relationship between the heater 1 power and the heater 1 current. Figure 8 illustrates the linear dependency of the heater 1 power on the heater 1 temperature. Figures 5–8 are discussed in more detail in §4.

### 3.2. The thermal influence on the ring absorption coefficient and the effective index

**Figure 9.**
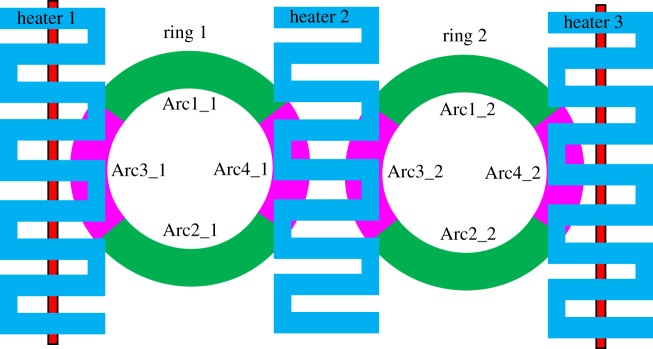
Partitioning of the rings into arcs.

As the effective index and group effective index are influenced by the proximity of the coupling waveguides and by the heater current and temperature, the resonator model was partitioned into arcs as shown in figure 9. The effective and group effective indices of the arcs were determined using the FemSIM thermo-optic multi-physics software.

Arc_1_, Arc_2_, etc., are the lengths of the arc segments.

The phase accumulated along a single arc (e.g. Arc_1_) = *jk*_0_*n*_eff_Arc_1_. Here *k*_0_ is the wave propagation vector and *n*_eff_ is the effective index. Since the waveguides are dispersive, the group effective index is used in the MATLAB modelling program.

The total transmission around each ring is

3.3 $$K_{\mathrm{total}}=K_{1}\times K_{2}\times K_{3}\times K_{4},$$

where *K*_1_, *K*_2_, *K*_3_ and *K*_4_ are the intensity transmissions for each arc section of the rings.

Substituting the relevant values into equation (3.3) gives

3.4 $$\begin{array}{rl} K_{\mathrm{total}} & ={\text{e}}^{\left(-\alpha {\mathrm{SiO}}_{2}/2)({\mathrm{Arc}}_{1}+{\mathrm{Arc}}_{2}\right)}\times {\text{e}}^{\left(-\alpha {\mathrm{SiO}}_{2}/2)({\mathrm{Arc}}_{3}+{\mathrm{Arc}}_{4}\right)} \end{array}$$

3.5 $$\begin{array}{rl} K_{\mathrm{total}} & ={\text{e}}^{-(\alpha {\mathrm{SiO}}_{2}/2)({\mathrm{Arc}}_{1}+{\mathrm{Arc}}_{2})+(\alpha {\mathrm{SiO}}_{2}/2)({\mathrm{Arc}}_{3}+{\mathrm{Arc}}_{4})}, \end{array}$$

where *α*SiO_2_ is the ring absorption coefficient.

The group effective index, effective index and ring transmission loss were calculated using the data generated by the FEM and BPM software. All effective and group effective index data for heater 1 currents are shown in tables 9–12 of appendix A1.

### 3.3. Ambient temperature change and thermal feedback

The ambient temperature in the COMSOL 3D model was varied to determine the effect on the waveguide core temperature. A parametric sweep of the ambient temperature, varied over the inclusive range of 263.15 –313.15 K, was applied to the 3D model at two central heater currents of 6 and 7 mA. Tables 5 and 6 show the data of these simulations.

**Table 5.** Waveguide core temperature change for ambient temperature change (heater current = 6 mA).

ambient temperature	waveguide core temperature	central heater temperature
263.15	303.8	375.2
273.15	313.8	385.2
283.15	323.8	395.2
293.15	333.8	405.2
303.15	343.8	415.2
313.15	353.8	425.2

**Table 6.** Waveguide core temperature change for ambient temperature change (heater current = 7 mA).

ambient temperature	waveguide core temperature	central heater temperature
263.15	318.5	415.7
273.15	328.5	425.7
283.15	338.5	435.6
293.15	348.5	445.6
303.15	358.5	455.6
313.15	368.5	465.6

A change in the waveguide core temperature would also result in a thermal drift of the output spectrum. To illustrate how thermal feedback could be applied to reduce the effect of thermal drift in the resonator, consider a sensor ambient temperature change from 293.15 to 303.15 K for a central heater current of 7 mA. Since the ambient temperature increases, the waveguide core temperature also increases by approximately 10 K. The waveguide core temperature increase could be measured using an integrated thermal sensor and so the central heater current could then be reduced to 6 mA. This would result in a waveguide core temperature reduction to 343.8 K, thereby reducing the effect of the ambient temperature change.

## 4. Results and discussion

### 4.1. Sensor operation and simulation results

Both slotted ring resonators are (ideally) dimensionally and physically identical, and both resonators are subject to coherent excitation from a super-luminescent diode (SLD) source. The outer rings of each resonator are also separated by 900 nm and so it is assumed that there is a zero-field coupling between the two resonators. An increase in temperature of heater 1, over the input waveguide coupling region, increases the RI (as shown in figure 4*b*) of the input waveguide and partial ring waveguide below heater 1. The effective index and, therefore, the group effective index are also increased by the heater 1 temperature rise. This changes the phase of the light in the sense resonator with respect to the phase of the light in the reference resonator. The increase in the group effective index of the sense resonator causes an increase in the phase accumulation in the sense resonator with respect to the phase of the circulating field in the reference resonator. The phase difference between the sense and reference resonators results in an optical transparency being introduced into the combined output spectrum. The increase in refractive and effective index means that the waveguide confinement factor increases. There will be a higher field confinement within the input waveguide and part of the arcs of the slotted rings which are influenced by the heater. As there will be less power transferred to the rings due to the higher field confinement of the input waveguide, more power will be transferred to the output through port. It is clear that because there will be more power in the output through port waveguides, the coupling coefficient will decrease and this in turn will increase the *Q* of the resonator (see tables 7 and 8 for FWHM and resonator *Q*). The combined output spectrum *Q* will also be reduced if the two resonators are not perfectly matched. The application of a temperature increase to the rings also decreases the ring loss and this is evident from the data for the ring absorption coefficients of table 1. Table 1 also shows how the coupling coefficients decrease as the temperature increases. Since the coupling and ring loss are decreased, this will affect the FWHM and hence the *Q* and extinction ratios of the combined output spectra. As both resonators are (ideally) dimensionally matched and are in close proximity, then both resonators are subject to the same ambient temperature rise and structural change, so the resonant peaks of both resonators will shift by the same amount. Only the differential spectral distance between the reference peak and the stimulus induced sense peak and extinction ratios needs to be measured.

**Table 7.** Ring characteristics with and without heater current applied.

extinction ratio at λ_res_ = 1.55139 µm	0.9711 − 0.0043 = 0.9668
free spectral range	1.55568 − 1.55139 µm = 4.29 nm
FWHM (no heater currents applied (unperturbed))	439 pm (*Q* = 3534)
FWHM (central heater current = 9 mA)	383 pm (*Q* = 4064)

**Table 8.** Spectral characteristics of the thermally induced optical transparency.

heater 1 current	thermally induced spectral shift of optical transparency	FWHM of transparency
7 mA	1.55318 − 1.55139 µm = 1.79 nm	225 pm (*Q* = 6903)
9 mA	1.55445 − 1.55139 µm = 3.06 nm	172 pm (*Q* = 9038)

If thermal drift compensation is a requirement in this design, then applying thermal feedback, as outlined in §3.3 to the central heater, could provide a reduction in the thermal drift of the output spectrum. Tables 5 and 6 show the change in waveguide and central heater temperatures for a change in the sensor ambient temperature. An illustration of applying thermal feedback to the central heater is provided along with the tables. The latter illustration is a coarse example of applying thermal feedback which results in bringing the waveguide core temperature back towards a quiescent operating temperature. With the integration of a temperature sensing element and supporting electronics on the chip, a finer control of the central heater current can be achieved.

There is a distinct difference between how the optical transparency is obtained in the OTRR spectrum and how the optical transparency is realized using the CRIT. In the CRIT configuration, the optical transparency is a function of the coupling strength between the two coupled resonators. In the OTRR, however, the optical transparency is realized by the influence of the heater temperature on the input and ring waveguides. The position of the optical transparency in the output spectrum is a function of the group effective index of the sense resonator rings while the extinction ratio is a function of the input waveguide-to-ring coupling coefficient. It should also be noted that the method of obtaining the optical transparency in the OTRR does not result in a Fano type resonance as shown for the CRIT (fig. 3 of [1]). The CRIT introduces a resonance split to both sides of a central resonant wavelength as shown in figure 11. In the CRIT, the central resonant wavelength without a split is realized by an optimized coupling distance between the two rings.

### 4.2. Simulation results

The simulation data show a high degree of correspondence between the temperature distributions in both the 2D and the 3D models. Table 2 compares the 2D and 3D ring waveguide core temperatures. It is clear from table 2 that there is very little difference in the ring waveguide core temperatures of the 2D and 3D models. A high level of confidence in the 2D heater temperature distribution data can therefore be assumed in order to obtain the temperature-dependent dispersion data for the subsequent spectral plots. A full current density parameter sweep, using incremental current density steps of 1.11 × 10^9^A m^−2^, was undertaken in the 3D COMSOL model. A heater current density, corresponding to 7 mA heater current was chosen to display the thermal response of the model in terms of the contour and waveguide core profile plots of figures 3 and 4.

Figure 3*a* illustrates how the heat is distributed around the heater and this contour colour plot shows that the heat is directed vertically (mostly) downwards toward the waveguides. There is relatively little heat transport in the lateral direction and this is a consequence of the geometry and dimensions of the heater. It can be seen from figure 3*b*, that for a heater current of 7 mA, with a corresponding heater temperature of approximately 443 K, the waveguides are subject to an 80 K temperature increase above the substrate ambient. The absolute temperature of 373 K, of the waveguide core, shows how the low thermal conductivity of SiO_2_ affects the transfer of heat from the heater to the waveguides. Figure 4*a* shows the RI change as a colour intensity profile. This colour contour plot shows that the highest RI change is in the central waveguide position directly beneath the heater. Figure 4*b* also shows the greatest RI increase is in the waveguides and also illustrates the relatively low RI change for the SiO_2_ material.

A 7 mA heater 1 current was chosen from the full parametric current range because the spectral position of the optical transparency, at this heater current, was near the mid-point in the unperturbed free spectral range. If the incremental RI data in table 3, obtained using the FemSIM multi-physics utility, is compared to the empirically determined incremental RI data of table 4, it can be seen that there are errors between the two sets of RI change data. The FemSIM derived change in refractive index (ΔRI) is 0.0147 for a 7 mA heater 1 current, where the empirically derived ΔRI is 0.0163 for the same simulated heater current. The FemSIM multi-physics simulation software utility uses a constant thermo-optic coefficient of 1.81  × 10^−4^ K^−1^ (input by the user) in the generation of the thermal data and does not appear to consider the thermo-optic coefficient as a function of temperature. If a constant value for the thermo-optic coefficient of 1.81 × 10^−4^ K^−1^ is used for each incremental temperature, then we arrive at the RI data in table 3. This latter observation shows how inaccuracies can be introduced into the simulated model. Figure 5 shows that the coefficient of linear expansion is two orders of magnitude smaller than the thermo-optic coefficient of silicon shown in figure 6. As a consequence, the linear expansion coefficient term in equation (3.1) may be omitted in determining Δλ_res_. Figures 5 and 6 confirm that, at the maximum heater current of 13 mA, the linear expansion coefficient would have a negligible influence on the resonant wavelength shift as determined by applying equation (3.1). The dominant factor in equation (3.1) is therefore the thermo-optic gradient of the effective index of the silicon rings. Figures 7 and 8 illustrate the nonlinear and linear dependency of the heater power to heater current and heater power to heater temperature, respectively. These figures also show the maximum heater temperature (approx. 812 K) for a maximum current of 13 mA in the TiN heater.

This heater temperature is well below the specified melting point of TiN (3243.15 K) [12]. The data of figures 7 and 8 could be used as a means of auto-calibration if the sensor is used in one of the suggested applications outlined in §5.

Simulations also established a minimum heater-to-waveguide distance such that the TiN heaters would not have a significant influence on the propagating modes of the E-field. The RI of TiN [13] is approximately 2.921 at 1.55 μm, which is higher than the RI of the silicon dioxide buffer layer. The absorption coefficient of TiN is also extremely high (approx. 437 × 10^3^ cm^−1^ [13]). It is, therefore, clear that a TiN component near to the silicon waveguide would unduly influence the mode field propagating through the waveguides beneath the heater. A heater-to-waveguide distance, where the TiN heater had a negligible effect on the waveguide propagating E-field, was determined to be 1.2 µm.

The OTRR transfer function, shown in equation (4.1), was derived using Mason's Rule.

The transfer function for the combined through port is

**Figure 10.**
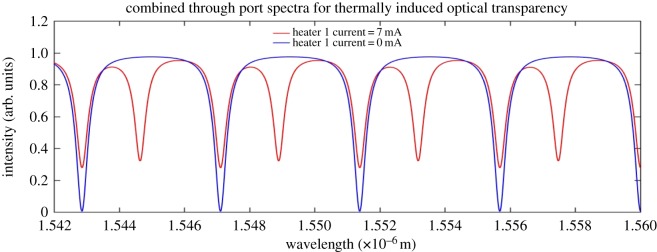
Combined through port output spectra of the OTRR.

**Figure 11.**
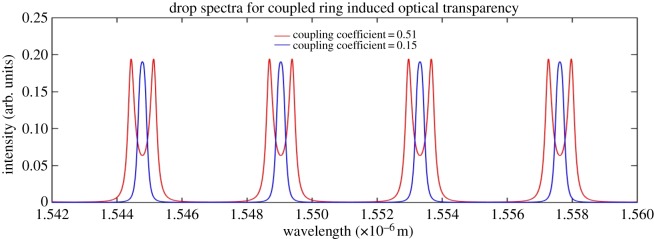
Coupling induced resonant split (no simulated heaters applied in this model.

**Figure 12.**
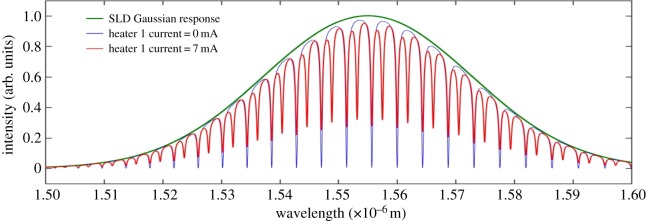
Combined through port spectral output for a 7 mA heater 1 current.

**Figure 13.**
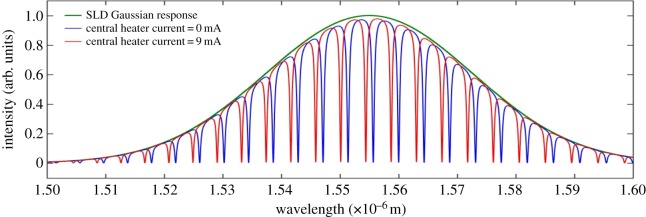
Combined through port spectral output for a 9 mA central heater current.

**Figure 14.**
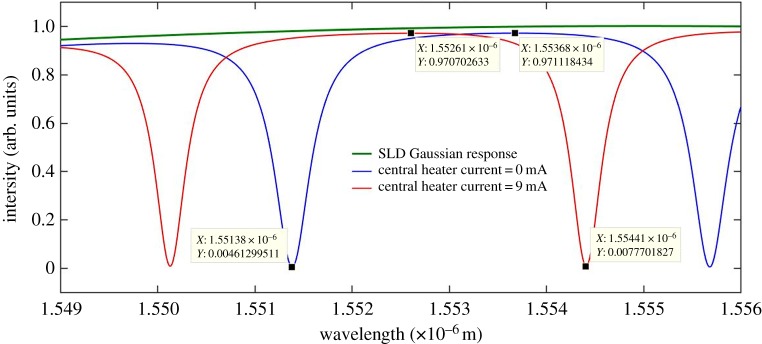
Expanded spectrum of of figure 13.

**Figure 15.**
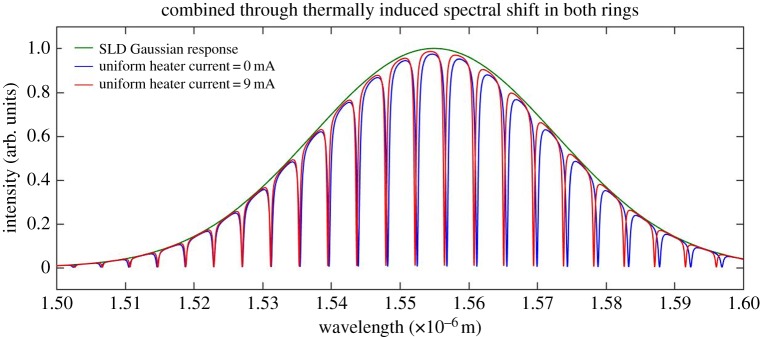
Combined through port spectral output for a 9 mA uniform heater current.

**Figure 16.**
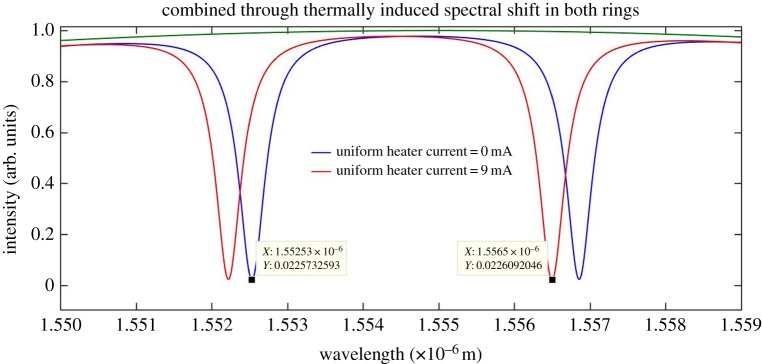
Expanded spectrum of figure 15.

4.1 $$\begin{array}{rl} E_{\mathrm{tcomb}} & =\left[\frac{E_{\mathrm{in}1}[t_{1}-t_{2}a_{1}e_{1}-t_{1}t_{2}\;t_{3}a_{2}e_{2}+{\left(t_{2}\right)}^{2}t_{3}a_{1}e_{1}a_{2}e_{2}]}{1-t_{1}t_{2}a_{1}e_{1}-t_{2}t_{3}a_{2}e_{2}+t_{1}{\left(t_{2}\right)}^{2}t_{3}a_{1}e_{1}a_{2}e_{2}}\right]\cdots \\ & \;+\left[\frac{E_{\mathrm{in}3}[t_{3}-t_{2}a_{2}e_{2}-t_{1}t_{2}t_{3}a_{1}e_{1}+{\left(t_{2}\right)}^{2}t_{1}a_{1}e_{1}a_{2}e_{2}]}{1-t_{1}t_{2}a_{1}e_{1}-t_{2}t_{3}a_{2}e_{2}+t_{1}{\left(t_{2}\right)}^{2}t_{3}a_{1}e_{1}a_{2}e_{2}}\right] \end{array}\},$$

where *a*_1 _= exp(−*α*_1_*L*_1_/2); *e*_1_ = exp (−*jk*_0_*L*_1_*N*_1_); *a*_2 _= exp(−*α*_2_*L*_2_/2); *e*_2_ = exp(−*jk*_0_*L*_2_*N*_2_); *t*_1_, *t*_2_ are the through transmission coefficients for input guides one and two; *α*_1_, *α*_2_, the respective ring absorption coefficients; *k*_0_ = the free space wave vector; *N*_1_, *N*_2_, the respective ring effective index values (group effective index used for dispersive waveguides); *L*_1_, *L*_2_, the respective ring lengths. The data generated from the multi-physics and dispersion utilities were used in equation (4.1) to construct the spectral plots and these plots are presented as figures 10–16.

### 4.3. Functional difference between the OTRR and the CRIT resonator

Figures 10 and 11 show how the OTRR and the CRIT optical transparencies are realized in the output spectrum.

Figure 10 shows the introduction of the transparency (red spectrum) as a result of the phase difference between the sense and reference resonators. This figure shows that the extinction ratio of the transparency spectra is affected, due to a decreased coupling to the rings from the input waveguide, which is a consequence of the applied heater 1 current.

The phase difference in the sense resonator of the OTRR is a consequence of the heater 1 temperature influencing the group effective index of the rings beneath the heater. There is zero resonator-to-resonator coupling assumed in this model.

Figure 11 shows the spectral output of a CRIT resonator. It is evident from figure 11 that the resonant wavelength diverges from a central resonant wavelength (blue spectrum) which results in resonance splitting. The blue spectrum of figure 11 represents an ideally coupled CRIT resonator.

The splitting shown in figure 11 is induced by contra-rotating resonant modes producing a spectral division of the ideally coupled (blue) resonant peak. The differential spectral distance between the split resonant peaks is a function of the ring-to-ring coupling strength (coefficient). The optical transparency remains at the reference (blue) resonant wavelength. This is in contrast with figure 10 where the optical transparency is a result of a phase induced resonant wavelength shift from a reference wavelength.

If it were required to use the CRIT as a sensing device, the method to infer the stimulus causing the resonance change would require measuring the differential wavelengths of the induced split and/or to measure the amplitude of the minima of the split peak. Conversely, using the OTRR as a sensor, the stimulus can be inferred by measuring the shift in wavelength from the stationary reference wavelength and also to measure the change in the extinction ratio of the shifted resonant peak. Both the methods appear to acquire the same output data but the OTRR enables a less complex method of data acquisition.

When measuring the spectral change in the OTRR, there is only a requirement to measure the shifted peak spectral difference and the extinction ratio (assuming no thermal drift). In the CRIT sensor, however, it would be necessary to keep track and measure both the red and blue (long and short wavelength) shifted peaks as well as the amplitude of the resonant minima. The coupling coefficient in the CRIT (without thermal compensation) is also influenced by changes in the ambient temperature.

There will, therefore, be a double thermal noise contribution in the output spectrum of the CRIT. It could be argued that the OTRR is not exhibiting a resonant split but a resonant shift due to the sense resonator. Since the unperturbed spectrum is split, producing a smaller free spectral range, it is appropriate to use the term split when describing the thermally induced optical transparency. Potential sensing applications of the dual-ring configuration in this work are suggested and discussed in §5.

### 4.4 Output spectra of the OTRR and the influence of the broadband source bandwidth

The simulated excitation input to the OTRR is a broadband SLD. This source provides the coherent excitation to both rings over the required spectral range (approx. 1.525–1.575 µm). The SLD has an optical bandwidth of approximately 50 nm, centred at a wavelength of 1.555 µm. The effect of the SLD bandwidth must be considered to achieve a realistic output of the models. Assuming that a grating coupler would be used to transfer the SLD source into waveguides and rings, the grating coupler bandwidth must also be considered in modelling the output of the coupled resonator.

For simplicity a grating coupler design is assumed with a central wavelength of 1.555 µm and a bandwidth of 50 nm. Figures 12–16 show the influence of the SLD bandwidth on the expected simulated output. The SLD response is also included in these figures. Increasing the current in heater 1 results in a resonant peak shift in the sense resonator which, when combined with the unshifted peak of the reference resonator at the combined output, results in a transparency between the unperturbed free spectral range. The spectral distance between the transparency and the reference wavelength is therefore a function of the heater and waveguide temperature increase.

Figure 12 shows the position of the transparency of the OTRR with respect to the reference peak for a heater 1 current of 7 mA.

Figure 12 also shows how the spectral output is influenced by the spectral bandwidth of the SLD. It is also clear from figure 12 that the reference resonant peak is stationary.

A 9 mA current was applied to the central heater (heater 2) over the central region. The combined through port output for the 9 mA central heater current is shown in figure 13.

An expanded spectral section of figure 13 is shown in figure 14 which illustrates, with greater clarity, the spectral shift of the output of the combined through port. Amplitude figures are also given on the plot in order to determine the extinction ratio.

Figure 14 shows that there is very little change in the extinction ratio when the central heater is applied to both rings. Applying the central heater (heater 2) results in an equal spectral shift in both resonators, as both resonators are now subject to an equal amount of phase shift from the reference wavelength. A spectral shift of the whole spectrum may also be simulated by applying an equal current to both heater 1 and heater 3.

Applying a 9 mA heater current to the central heater gives a spectral shift in both resonators of

4.2 1.55441−1.55139 μm=+3.02 nm.

The plus sign in the above equation indicates a red (longer wavelength) spectral shift. The combined through port output, shown in figure 14, exhibits the positive red spectral shift of the equation. A transparency is not introduced into this output spectrum and as a consequence the free spectral range in the combined through port output remains unaltered. A uniform heat source beneath both resonators was simulated in the model in order to determine the amount of spectral shift given by this simulation and to compare this shift with the theoretically expected value given by equation (3.1). The latter simulation also allows for a comparison between the spectral shift of the uniform heater application and the spectral shift achieved by applying the heater to the partial arcs of the rings. The data generated by the uniform heater simulation are modelled in figure 15 and an expanded spectral section of figure 15 is given in figure 16 for greater clarity.

Figure 15 shows that a spectral shift, shown by the red spectrum, is positive with respect to the reference blue spectrum. To avoid ambiguity in interpreting the output spectra in the figures, it should be made clear that the blue spectrum does not appear at the output for the perturbed condition. It is shown only as an unperturbed reference spectrum.

A uniform heater current of 9 mA applied to both rings gives the positive spectral shift illustrated in figure 16.

Figure 16 gives an incremental spectral shift of

4.3 Δλ=1.5565−1.55253 μm=+3.97 nm, 

Δλ obtained from figure 16 can be compared with the value obtained using equation (3.1):

4.4 $$\Delta \lambda =\frac{\lambda_{0}}{n_{g0}}\frac{\partial n_{\mathrm{eff}}}{\partial T}\text{d}T,$$

d*T* = 133.24 K (increase in ring waveguide core temperature (from COMSOL data)); λ_0_ = unperturbed resonant wavelength = 1.55253 µm; *n*_g0_ = unperturbed group effective index = 3.587044

4.5 $$\frac{\partial n_{\mathrm{eff}}}{\partial T}=\frac{2.395340-2.360147}{520}=6.767\times 10^{-5}.$$

The *∂n*_eff_/*∂T* term was obtained from taking a parametric scan over temperature (table 13).

Inserting the relevant values into equation (4.4) gives a predicted spectral shift of

4.6 Δλ=+3.90 nm.

A spectral shift given by the above equation is a close approximation to the spectral shift shown in figure 16 and given by equation (4.3). This also results in a spectral sensitivity of 29 pm K^−1^ at a heater temperature of 541.8 K and a corresponding heater power of 92.1 mW. The spectral shift of equation (4.3) can now be compared with the spectral shift of equation (4.2). As expected, the spectral shift is larger for the uniform heater application than that where the central heater is applied only to the partial ring arcs.

The extinction ratio, FWHM, *Q* and the spectral shift data extracted from the plots of figures 14 and 16 are given in tables 7 and 8.

The application of the central or uniform heater exercises control of the quiescent, non-stimulus induced, spectral position of the reference wavelength. It should also be noted here that there is no optical transparency introduced when applying the central heater or a uniform heater and that the full spectrum red shifts as a function of increasing heater temperature.

## 5. Suggested sensor applications of the OTRR

There are two sensor applications suggested for this novel configuration *viz*.

(1) a gas mass flow sensor;
(2) a gas species identification sensor.

The suggested applications are briefly outlined in the following sections, and if viable, could be the subject of another paper. The two suggested applications require the integration of a micro-fluidics cell over the dual-ring arrangement.

A single micro-fluidics cell can be used in a flow mode for the mass flow sensor and a differential static cell could be integrated (or adapted from the static cell for multi-purpose sensing) for the gas species identification sensor.

### 5.1. Gas mass flow sensor

The central heater (heater 2) can be used as a partial heater to both rings while heaters 1 and 3 can be used as upstream and downstream differential flow sensors. The differential heater temperatures will be a function of the gas mass flow rate across the heaters. The combined output through ports should show that the spectral position of the optical transparency is a function of the differential temperatures of both heater 1 and heater 3 (upstream and downstream heaters) and hence this would infer the mass flow rate of the gas. Uniform heaters could alternatively be applied to both sets of rings by the integration of a buried heater beneath both ring resonators. If the latter heater integration method is adopted, care should be taken to ensure minimal interaction between the waveguide propagating field and the heater for the reasons given in §4.2.

### 5.2. Gas species identification sensor

The study of Hodgkinson & Tatam [14] gives an excellent insight into optical methods for the detection, measurement and characterization of a gas species. Hilfiker *et al.* [13] explores the technique of optical gas detection using absorption spectroscopy based on the Beer–Lambert Law. Another study [15] examines the use of a tungsten oxide-coated micro-ring resonator as a hydrogen sensor.

Vernier based micro-ring resonators have also been investigated [16] for the detection of gas and chemical elements. Particular attention should be paid to the intrinsic safety of any sensor design when designing a sensor that could potentially be used to detect or characterize flammable or volatile substances. Slotted micro-ring resonators have been investigated [17] in the detection of acetylene gas. In [17], the change in the RI of the gas is proportional to the change in the gas pressure and to the Gladstone–Dale constant at the interrogating wavelength. Based on the results of the OTRR simulations obtained in this paper, it is envisaged that a method which relates the properties of a gas species to the temperature of the gas could be inferred by the shift in the sense resonator wavelength. The spectral position and extinction ratio of the optical transparency of the combined through port would, therefore, be a function of the properties of the gas and could provide a gas species signature (species identification).

The latter may involve a complex multidisciplinary study and analysis of different heater materials which could be coated with gas-specific absorbent compounds such as doped tin oxide [18]. The study by Heilig *et al.* [18] describes the application of a modulated heat signal to a platinum heater and examines the thermal response of a tin oxide-coated sensor to identify a binary gas mixture of carbon monoxide and nitrogen dioxide.

## 6. Conclusion

The simulations and model outputs demonstrated in this paper introduce a novel method of achieving an optical transparency in a micro-ring resonator output spectrum. The thermally induced optical transparency realized in the OTRR is distinctly different to the optical transparency derived from the CRIT. The advantage of adopting the OTRR over the CRIT in introducing the transparency is that in the OTRR, a stationary reference wavelength as well as a stimulus induced dynamic wavelength (the transparency) is produced at the combined output port. The advantage of this is that the post-processing of the output data would be less complex than the post-processing required for the CRIT output data. Waveguide losses may be reduced by applying the shorter operating wavelength of 1.3 µm. Reducing ring and waveguide losses would enhance the resonator *Q* which could offer higher cross-channel immunity if the resonators were to be multiplexed in a sensor application. Two sensor applications are suggested for the OTRR, and in both applications, the rings and waveguides are not exposed to the gas species. This has the advantage in that the waveguide optical characteristics are not influenced by gas contaminants. The OTRR heaters could have the capability to auto-decontaminate by initiating a ‘burn-off' of gas contaminant residue which may have settled onto the heaters.

Since the ring waveguides are not exposed in the proposed applications, a redesign of the waveguide dimensions could be investigated, with a reduced operating wavelength, to minimize the waveguide losses. If the suggested OTRR applications were found to be viable then this could initiate further work in both of these application areas.

## 7. Further work

Variations on the novel design described in this paper could be adopted in order to simulate and fabricate a mass flow sensor or a sensor for the identification of a gas species. Further studies and investigations could be made into the type and suitability of gas-specific heater coatings. Computational fluid dynamics and heat transfer simulations could also be applied to the OTRR to determine the characteristics of heat transfer from the heater to the gases to be measured.

## Data accessibility

All data and MATLAB program files generated from the RSoft photonic simulation software are available at the Dryad Digital Repository http://dx.doi.org/10.5061/dryad.36n7v [19].

## Appendix A1 (figure 16)

See tables 9–12.

**Table 9.** Partial arc dispersion data (heater 1 current = 0 mA).

heater 1 current = 0 mA	effective index	group effective index
*ring 1*		
ARC 3_1	2.363069	3.558134
ARC1_1	2.357019	3.587044
ARC_2_1	2.357019	3.587044
ARC4_1	2.360720	3.563232
*ring 2*		
ARC 3_2	2.360720	3.563232
ARC1_2	2.357019	3.587044
ARC_2_2	2.357019	3.587044
ARC4_2	2.363069	3.558134

**Table 10.** Partial arc dispersion data (heater 1 current = 5 mA).

heater 1 current = 5 mA	effective index	group effective index
*ring 1*		
ARC 3_1	2.368179	3.567087
ARC1_1	2.357019	3.587044
ARC_2_1	2.357019	3.587044
ARC4_1	2.360720	3.563232
*ring 2*		
ARC 3_2	2.360720	3.563232
ARC1_2	2.357019	3.587044
ARC_2_2	2.357019	3.587044
ARC4_2	2.363069	3.558134

**Table 11.** Partial arc dispersion data (heater 1 current = 7 mA).

heater 1 current = 7 mA	effective index	group effective index
*ring 1*		
ARC 3_1	2.373096	3.574481
ARC1_1	2.357019	3.587044
ARC_2_1	2.357019	3.587044
ARC4_1	2.360720	3.563232
*ring 2*		
ARC 3_2	2.360720	3.563232
ARC1_2	2.357019	3.587044
ARC_2_2	2.357019	3.587044
ARC4_2	2.363069	3.558134

**Table 12.** Partial arc dispersion data (heater 1 current = 9 mA).

heater 1 current = 9 mA	effective index	group effective index
*ring 1*		
ARC 3_1	2.379656	3.585919
ARC1_1	2.357019	3.587044
ARC_2_1	2.357019	3.587044
ARC4_1	2.360720	3.563232
*ring 2*		
ARC 3_2	2.360720	3.563232
ARC1_2	2.357019	3.587044
ARC_2_2	2.357019	3.587044
ARC4_2	2.363069	3.558134

## Appendix A2

See tables 13 and 14.

**Table 13.** Effective index data for a rise in the ring waveguide temperature over the full ring.

waveguide core temperature	ring effective index for uniform heater
0.00 × 1	2.360147 × 1
2.50 × 10	2.361817 × 1
5.00 × 10	2.363483 × 1
7.50 × 10	2.365155 × 1
1.00 × 10^2^	2.366826 × 1
1.25 × 10^2^	2.368501 × 1
1.50 × 10^2^	2.370168 × 1
1.75 × 10^2^	2.371843 × 1
2.00 × 10^2^	2.373518 × 1
2.25 × 10^2^	2.375190 × 1
2.50 × 10^2^	2.376868 × 1
2.75 × 10^2^	2.378542 × 1
3.00 × 10^2^	2.380215 × 1
3.25 × 10^2^	2.381897 × 1
3.50 × 10^2^	2.383573 × 1
3.75 × 10^2^	2.385251 × 1
4.00 × 10^2^	2.386930 × 1
4.25 × 10^2^	2.388611 × 1
4.50 × 10^2^	2.390292 × 1
4.75 × 10^2^	2.391975 × 1
5.00 × 10^2^	2.393655 × 1
5.25 × 10^2^	2.395340 × 1

**Table 14.** Thermal, current, voltage and power heater data determined from the application of the 3D COMSOL model.

heater 1 current density (A m^−2^)	heater current (A)	heater voltage (V)	heater power (W)	heater temperature (K)
1.11 × 10^9^	1.00 × 10^−3^	1.14	1.14 × 10^−3^	296.2197
2.22 × 10^9^	2.00 × 10^−3^	2.27	4.54 × 10^−3^	305.4288
3.33 × 10^9^	3.00 × 10^−3^	3.41	1.02 × 10^−2^	320.7773
4.44 × 10^9^	4.00 × 10^−3^	4.55	1.82 × 10^−2^	342.2653
5.56 × 10^9^	5.00 × 10^−3^	5.68	2.84 × 10^−2^	369.8926
6.67 × 10^9^	6.00 × 10^−3^	6.82	4.09 × 10^−2^	403.6593
7.78 × 10^9^	7.00 × 10^−3^	7.96	5.57 × 10^−2^	443.5654
8.89 × 10^9^	8.00 × 10^−3^	9.1	7.28 × 10^−2^	489.6109
1.00 × 10^10^	9.00 × 10^−3^	10.23	9.21 × 10^−2^	541.7958
1.11 × 10^10^	1.00 × 10^−2^	11.37	1.14 × 10^−1^	600.1201
1.22 × 10^10^	1.10 × 10^−2^	12.51	1.38 × 10^−1^	664.5837
1.33 × 10^10^	1.20 × 10^−2^	13.64	1.64 × 10^−1^	735.1867
1.44 × 10^10^	1.30 × 10^−2^	14.78	1.92 × 10^−1^	811.929
